# The electronic structure of K_2_SiF_6_ from GW calculations and photoelectron spectroscopy

**DOI:** 10.1039/d6tc01537a

**Published:** 2026-07-17

**Authors:** Uday Kushwah, (Ann) Shiyang Lu, Prajna Bhatt, Aysha A. Riaz, Pardeep K. Thakur, Tien-Lin Lee, Marco Kirm, Vitali Nagirnyi, Tanel Käämbre, Johannes Lischner, Anna Regoutz, Juhan Matthias Kahk

**Affiliations:** a Institute of Physics, University of Tartu W. Ostwaldi Str 1 50411 Tartu Estonia juhan.matthias.kahk@ut.ee; b Departments of Materials and the Thomas Young Centre for Theory and Simulation of Materials, Imperial College London London SW7 2AZ UK j.lischner@imperial.ac.uk; c Istituto Officina dei Materiali (IOM)-CNR, in Area Science Park, S.S.14, Km 163.5 Trieste I-34149 Italy; d Department of Chemistry, University of Oxford, Inorganic Chemistry Laboratory South Parks Road Oxford OX1 3QR UK; e Diamond Light Source Ltd., Diamond House, Harwell Science and Innovation Campus Didcot OX11 0DE UK

## Abstract

Ultrafast scintillators based on ternary hexafluorides are promising for next-generation radiation detectors, which can be used in time-of-flight positron emission tomography. To gain a detailed understanding of the scintillation mechanism in these materials, accurate knowledge of the electronic band structure is required. In this study, photoelectron spectroscopy, density-functional theory, and G_0_W_0_ calculations were used to investigate the electronic structure of K_2_SiF_6_. The G_0_W_0_ calculations predict a wide band gap of 12.4 eV reflecting the strongly ionic character of the bonding. In contrast to predictions from semi-local or hybrid density-functional theory calculations, the large band gap predicted by G_0_W_0_ suggests that Auger–Meitner decay of K 3p holes is energetically not allowed and that scintillation *via* cross-luminescence is possible in this material. However, the poor light-yield observed experimentally indicates that the exclusion of Auger–Meitner decay is not sufficient for good scintillation performance, and cross-luminescence competes with other decay channels.

## Abstract 2

An investigation of the electronic structure of K_2_SiF_6_ based on GW theory and photoelectron spectroscopy reveals that Auger–Meitner decay of K 3p holes is energetically forbidden in this material.

## Graphical abstract

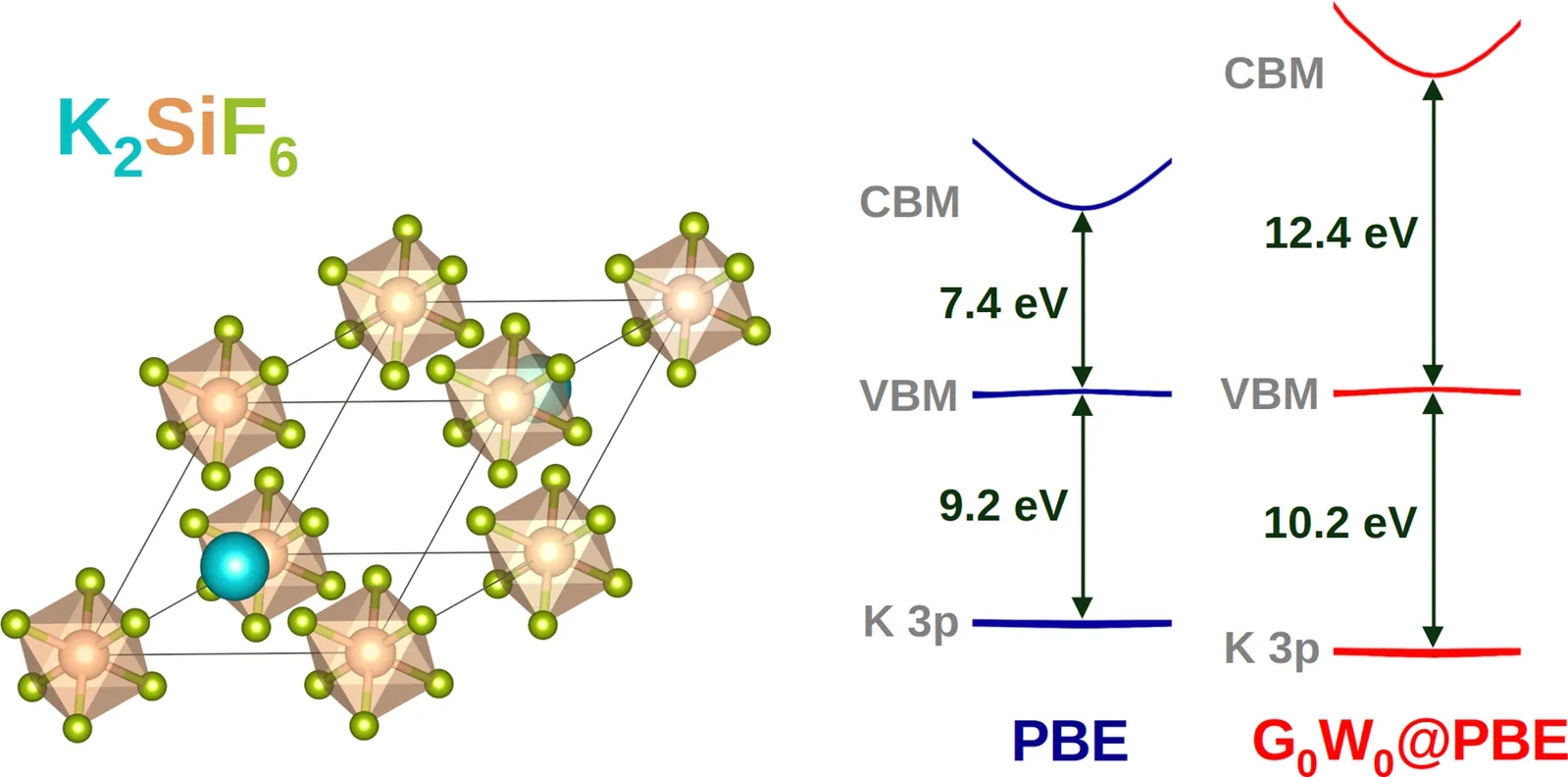

## 1 Introduction

The performance of scintillator detectors is one of the key factors that determine the quality of recorded images in X-ray and gamma-ray based medical imaging devices.^1–3^ In particular, in positron emission tomography (PET), the signal-to-noise ratio of the image depends on the ability of gamma-ray detectors to correctly identify coincidence events in which two gamma-rays are simultaneously emitted following positron–electron annihilation inside the patient's body.^4,5^ Effective scintillators should have high light yield and ultrafast emission and decay to avoid the detection of false coincidences.^6,7^ Moreover, in time-of-flight PET, sub-nanosecond response time of the scintillator can further improve image quality by permitting the localization of the annihilation event along the line of coincidence.^1,5,8^

Previously, it has been shown that the binary fluoride BaF_2_ exhibits an ultra-fast scintillation response in the form of cross-luminescence – the emission of light due to electronic transitions from the F 2p valence states to the Ba 5p semi-core band – with a decay time of ≈0.9 ns.^9^ However, BaF_2_ has several shortcomings that hinder its practical use in radiation detection: the low light-yield of the ultrafast decay channel, the fact that the ultrafast decay leads to emission in the deep UV region, and the presence of a competing excitonic decay channel with a higher light yield and a slow decay time.^10–12^

To overcome these limitations, several groups have searched for alternative inorganic solids that have the potential to exhibit ultrafast cross-luminescence. Ternary fluorides A_*m*_B_*n*_F_*k*_, where A is a group I or group II element and B is typically Si or a metal or metalloid with a d^0^ or d^10^ electronic configuration, have attracted considerable attention because of the possibility of using band structure engineering by modifying the composition to increase the light yield and to reduce the photon energy of the emitted light.^8,10,12,13^ For example, Saaring *et al.*^8^ studied the scintillation properties of pure K_2_SiF_6_. They observed that the material exhibits several cross-luminescence bands in the region of 4 to 9 eV, originating from radiative recombination between a hole in the K 3p semi-core band and a valence electron. Time-resolved measurements further demonstrated that these cross-luminescence bands possess exceptionally fast sub-nanosecond decay components, with characteristic times of ∼80 ps and ∼230 ps, significantly faster than the observed decay times in BaF_2_. Despite this ultrafast response, the overall cross-luminescence scintillation yield was found to be modest (∼25 ph per MeV). The ultrafast scintillation response of K_2_SiF_6_ would make it a prime candidate for scintillator detectors, but the low light yield is a serious problem for practical applications. It is therefore desirable to understand the factors that determine the scintillation response of this material.

From an electronic structure perspective, processes like cross-luminescence and intraband luminescence – the emission of light due to transitions within the valence (or conduction) manifold – depend on the delicate balance between radiative and non-radiative decay channels of holes in semi-core and valence states, or the decay channels of excited electrons in the conduction band. For example, for cross-luminescence, it is believed to be crucial that Auger–Meitner decay of the semi-core hole should be energetically forbidden.^9,14^ In order to make computational predictions about new scintillator materials, calculations that can accurately describe the energies of both the occupied and empty bands are therefore required.^15^

First principles methods find widespread use in the design and optimization of new optoelectronic materials.^16–21^ At present, the electronic structures of K_2_SiF_6_ and similar materials have been mostly studied using density functional theory (DFT). For example, Brik and Srivastava^22^ performed DFT calculations of K_2_SiF_6_ based on the local density approximation (LDA) and the generalized gradient approximation (GGA) with the Perdew–Burke–Ernzerhof (PBE) functional. A direct band gap of 7.6 eV (GGA) and 8.1 eV (LDA) was reported at the Γ point.^23^ These values were acknowledged as underestimations of the true fundamental gap as a consequence of the well-known limitations of semi-local exchange–correlation functionals. The calculated band structure exhibited very flat valence bands and more dispersive conduction bands, in agreement with subsequent results obtained by Zhao *et al.*^24^ In a follow-up study, several global hybrid functionals with a fixed fraction of exact exchange were used to compute the electronic structure. Among these, the B1WC functional produced the largest band gap of 9.73 eV.^25^ However, it is known that general purpose hybrid functionals also systematically underestimate band gaps in wide band gap insulators.^26,27^ Even for the occupied states, Kohn–Sham eigenvalues are not always a reliable estimate of measured quasiparticle energy levels.^28^

In this work we have performed a more detailed investigation into the electronic structure of K_2_SiF_6_ using a combination of experimental and theoretical tools, namely photoelectron spectroscopy (PES) and many-body perturbation theory (MBPT) in the G_0_W_0_ formalism. The agreement between the experimental and computational results is evaluated, the electronic structure of K_2_SiF_6_ is analyzed by comparing the GW results to the predictions of a simple model based on symmetry considerations, and the results are discussed in terms of the general requirements for ultrafast scintillators.

## 2 Methods

DFT calculations were carried out using the Quantum Espresso software package,^29^ employing the generalized gradient approximation with the PBE exchange–correlation functional together with optimized norm-conserving Vanderbilt pseudopotentials.^23,30^ A plane-wave kinetic energy cutoff of 60 Ry and Brillouin zone sampling with an 8 × 8 × 8 Monkhorst–pack grid were used for all calculations.

The structural parameters of K_2_SiF_6_ were determined through a series of fixed-cell relaxations in which the cubic lattice parameter was varied between 5.250 Å and 6.126 Å in 16 steps. For each cell volume the atomic positions were relaxed using the Broyden–Fletcher–Goldfarb–Shanno (BFGS) algorithm. The equilibrium lattice constant was obtained from the minimum of the resulting energy–volume curve.

Next, self-energy corrections to the DFT eigenvalues were obtained using many-body perturbation theory. Calculations were performed within the G_0_W_0_ approximation using the BerkeleyGW software package.^31,32^ The frequency dependence of the dielectric matrix was treated using the Hybersten–Louie generalized plasmon-pole approximation.^33^ The entire set of G_0_W_0_ calculations was repeated using three different values of the plane-wave cutoff for the dielectric matrix: 30 Ry, 35 Ry, and 40 Ry. For each value of the plane-wave cutoff, the number of bands included in the calculation of the dielectric matrix was chosen such that the DFT eigenvalue of the highest included band, referenced to the VBM at zero, was approximately equal to the cutoff energy. As such, 2709, 3408, and 4094 total bands (occupied and empty) were used at 30 Ry, 35 Ry, and 40 Ry, respectively. Subsequently, for each plane-wave cutoff, the eigenvalues were calculated – the summation over empty states was carried out using 2708, 3407, and 4093 bands for the three cutoffs, and the static remainder correction was used in each case.^34^ Finally, the obtained quasiparticle energies were extrapolated to the infinite-cutoff limit using a linear fit extrapolation method.^35^

A separate set of DFT calculations was performed for the Bader charge analysis, employing projector-augmented wave (PAW) pseudopotentials from the PS Library^36^ to obtain the all electron charge density. In these calculations the plane-wave cut-off was 80 Ry. The Brillouin zone was sampled with a 36 × 36 × 36 Monkhorst–Pack grid. The resulting charge density was written in cube format on an 80 × 80 × 80 uniform real-space grid, and Bader partitioning was carried out using the Critic2 code.^37^

K_2_SiF_6_ microparticles were synthesized *via* microwave-hydrothermal treatment – the details of the synthesis and the characterization of the resulting material using X-ray diffraction and electron microscopy have already been reported in Vanetsev *et al.*^38^

Laboratory-based X-ray photoelectron spectroscopy (XPS) data were collected on a Thermo Scientific NEXSA G2 XPS system at the EPSRC National Facility for XPS, Harwell, UK, with a monochromated, microfocused Al K_α_ X-ray source with a photon energy, *hν*, of 1486.7 eV, and had a spot size of 400 µm. Key core level and valence band spectra were collected using 20 eV and 15 eV pass energy, respectively. The samples were mounted on conducting carbon tape, and a dual Ar^+^ ion and electron source flood gun was used to compensate for sample charging. Synchrotron-based hard X-ray photoelectron spectroscopy (HAXPES) was performed at beamline I09 at the Diamond Light Source, Harwell, UK. A photon energy of 5.927 keV was used to perform the measurements, which will be further referred to as 6 keV for simplicity. The analysis chamber of the beamline end station has a base pressure of ∼3.5 × 10^10^ mbar and measurements were performed in grazing incidence and near normal emission geometry to allow the efficient collection of spectra. Core level and valence band spectra were collected using a pass energy of 200 eV. For both SXPS and HAXPES, all spectra were aligned to the adventitious carbon peak in the C 1s spectra at a binding energy (BE) of 284.8 eV. The CasaXPS software package was used to determine the total F 1s and VB areas, which were used to normalise all core level spectra, and the valence band spectrum, respectively.

## 3 Results

**Fig. 1:**
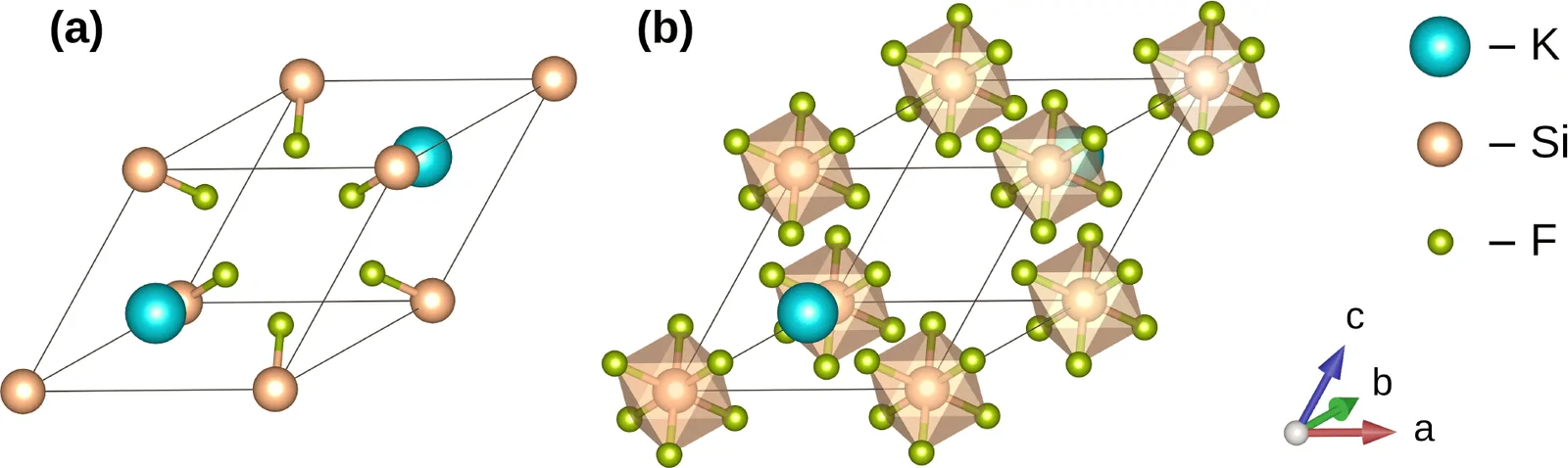
Crystal structure of K_2_SiF_6_ (space group *Fm*3̄ *m*): (a) primitive unit cell; (b) [SiF_6_]^2−^ octahedra embedded in a lattice of K^+^ cations.

K_2_SiF_6_ is a wide band gap insulator that crystallizes in the K_2_PtCl_6_-type structure with space group *Fm*3̄*m* (No. 225). The experimental lattice parameter is 8.142 Å for the conventional cell^39^ or equivalently 5.757 Å for the primitive cell. Silicon atoms occupy the 4a Wyckoff sites and are octahedrally coordinated by six fluorine atoms, forming isolated [SiF_6_]^2−^ units. Potassium atoms reside on the 8c sites and are coordinated by twelve fluorine neighbours, while fluorine atoms occupy the 24e sites and are bonded to one Si and four K atoms. The resulting lattice can be viewed as a framework of K^+^ cations surrounding weakly interacting [SiF_6_]^2−^ octahedra, consistent with the formal oxidation states K^+^, Si^4+^, and F^−^. Geometry optimization of K_2_SiF_6_ was performed using DFT with the PBE functional, as described in the methods section. The relaxed lattice parameter is 5.834 Å, which is within 1.4% of the experimental value and almost identical to the value reported in the AFLOW database,^40^ also based on PBE calculations. The relaxed structure was used for all subsequent electronic structure calculations. The primitive unit cell of K_2_SiF_6_ is shown in Fig. 1, and the atomic positions in the relaxed primitive cell are listed in Table 1.

**Table 1:** Atomic positions of K_2_SiF_6_ in the primitive unit cell in fractional coordinates, for the relaxed structure

Atom	*x*	*y*	*z*
Si	0.000	0.000	0.000
K	0.250	0.250	0.250
K	0.750	0.750	0.750
F	0.791	0.209	0.209
F	0.209	0.791	0.791
F	0.791	0.791	0.209
F	0.209	0.209	0.791
F	0.209	0.791	0.209
F	0.791	0.209	0.791

**Fig. 2:**
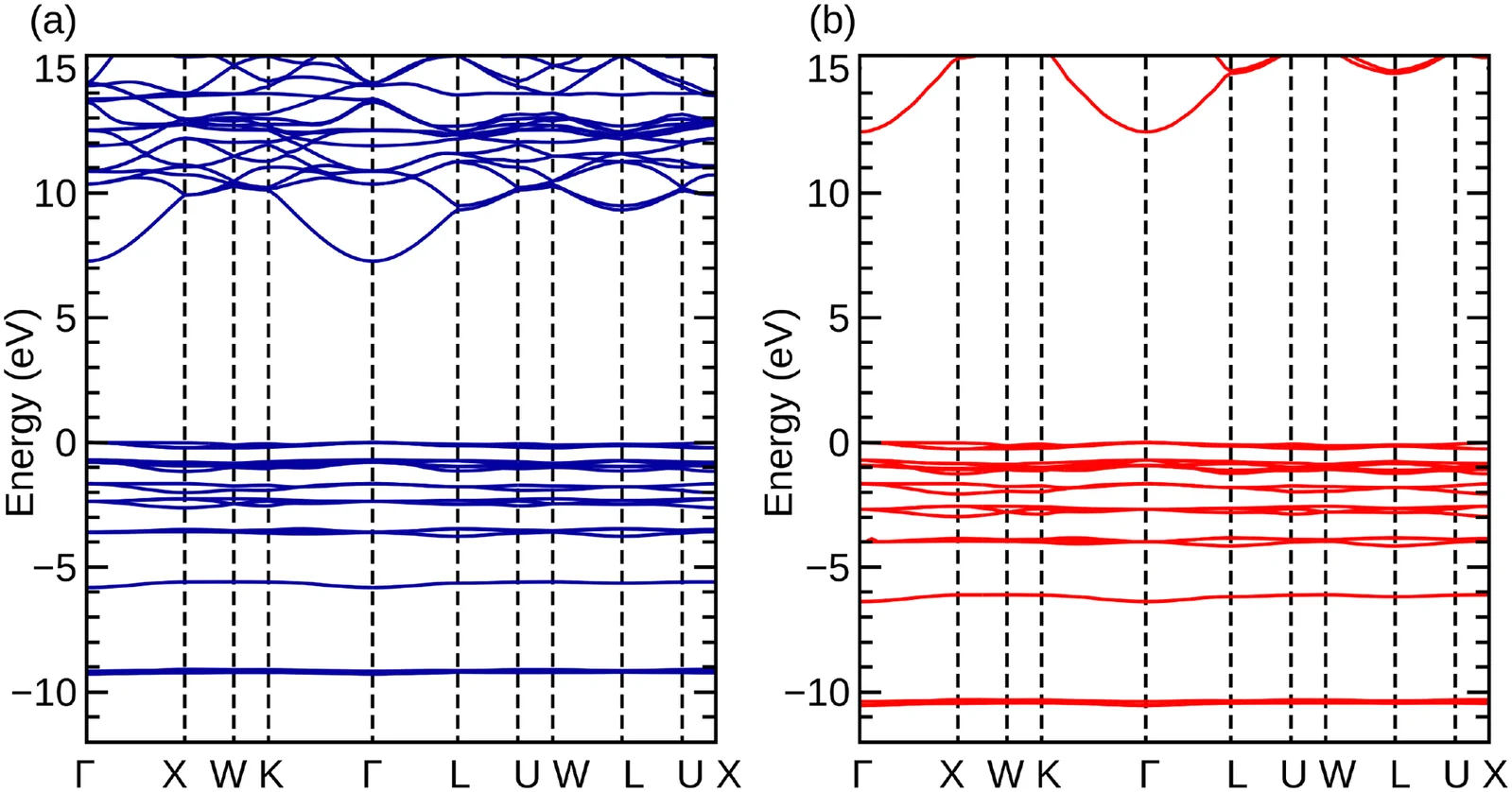
Electronic band structure of K_2_SiF_6_ obtained using (a) PBE, and (b) G_0_W_0_@PBE. Energies are referenced to the valence band maximum at 0 eV.

The calculated band structures of K_2_SiF_6_ from the PBE and G_0_W_0_@PBE calculations are shown in Fig. 2. In both panels, the zero of the energy scale has been aligned with the valence band maximum (VBM) at 0 eV. In agreement with previous results, we find the valence bands to be very flat (both in PBE and in G_0_W_0_), with band widths on the order of a few tenths of an eV, whereas the conduction bands are highly dispersive, with band widths of several eV. The main effect of the G_0_W_0_ correction is to increase the size of the direct band gap at Γ to 12.4 eV, which is significantly larger than the 7.4 eV predicted by PBE, and also larger than the band gaps of ∼10 eV predicted by regular hybrid functionals.^25^ Such large quasiparticle gaps are characteristic of highly ionic fluorides, which exhibit weak dielectric screening and correspondingly large exciton binding energies.^41–44^ As a result, hybrid functionals and optical absorption measurements tend to systematically underestimate the fundamental band gap in these materials. We also found that a one-shot hybrid DFT calculation with PBEh, based on the charge density from PBE, employing 43% of exact exchange reproduced the G_0_W_0_ band gap.

**Fig. 3:**
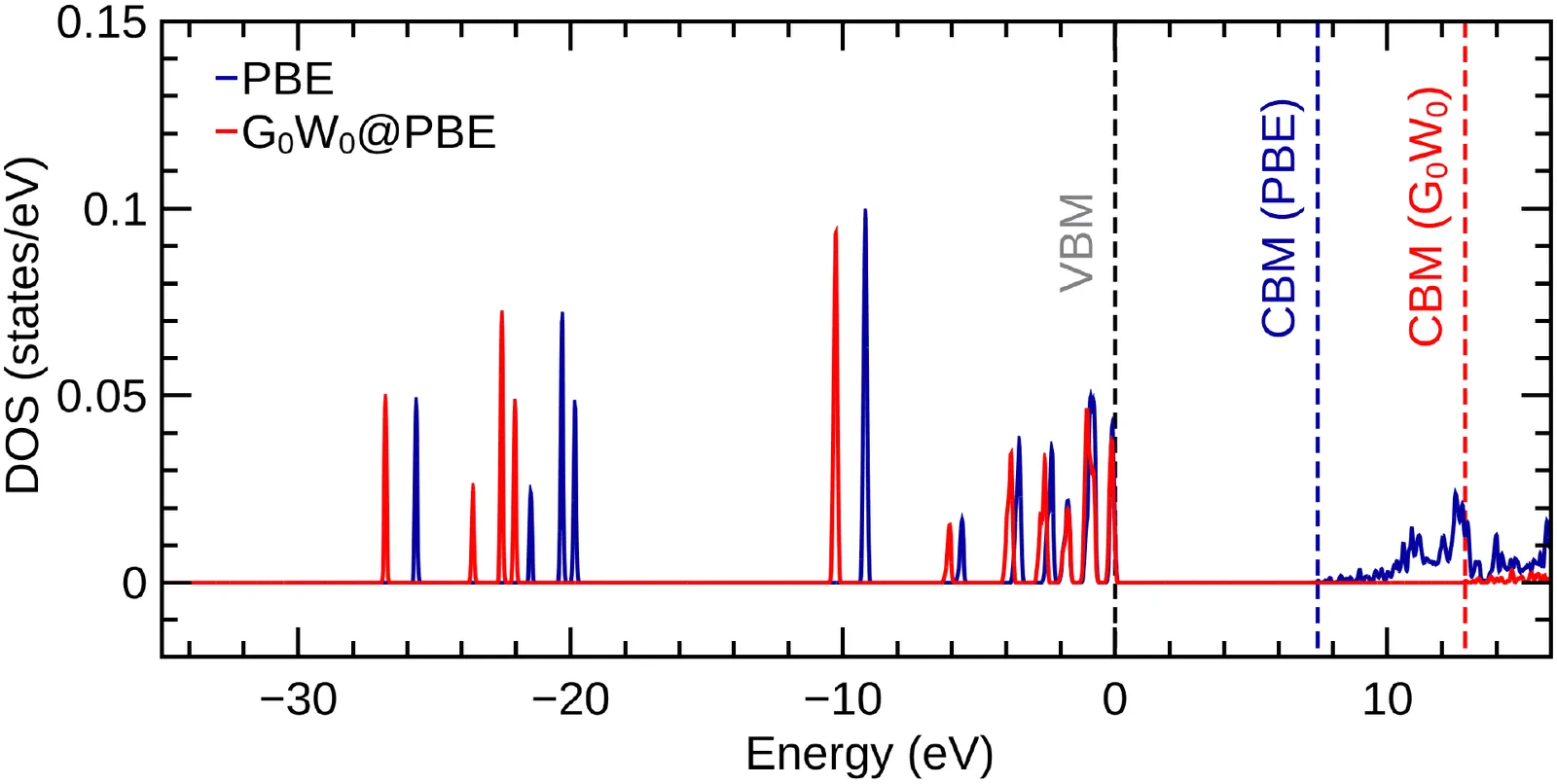
Total density of states (DOS) of K_2_SiF_6_ calculated using PBE (blue) and G_0_W_0_@PBE (red), aligned to the valence band maximum (VBM) at 0 eV. The grey dashed vertical lines indicates the position of the VBM and the blue and red dashed lines indicate the positions of the conduction band minimum (CBM) from PBE and G_0_W_0_@PBE, respectively.

**Fig. 4:**
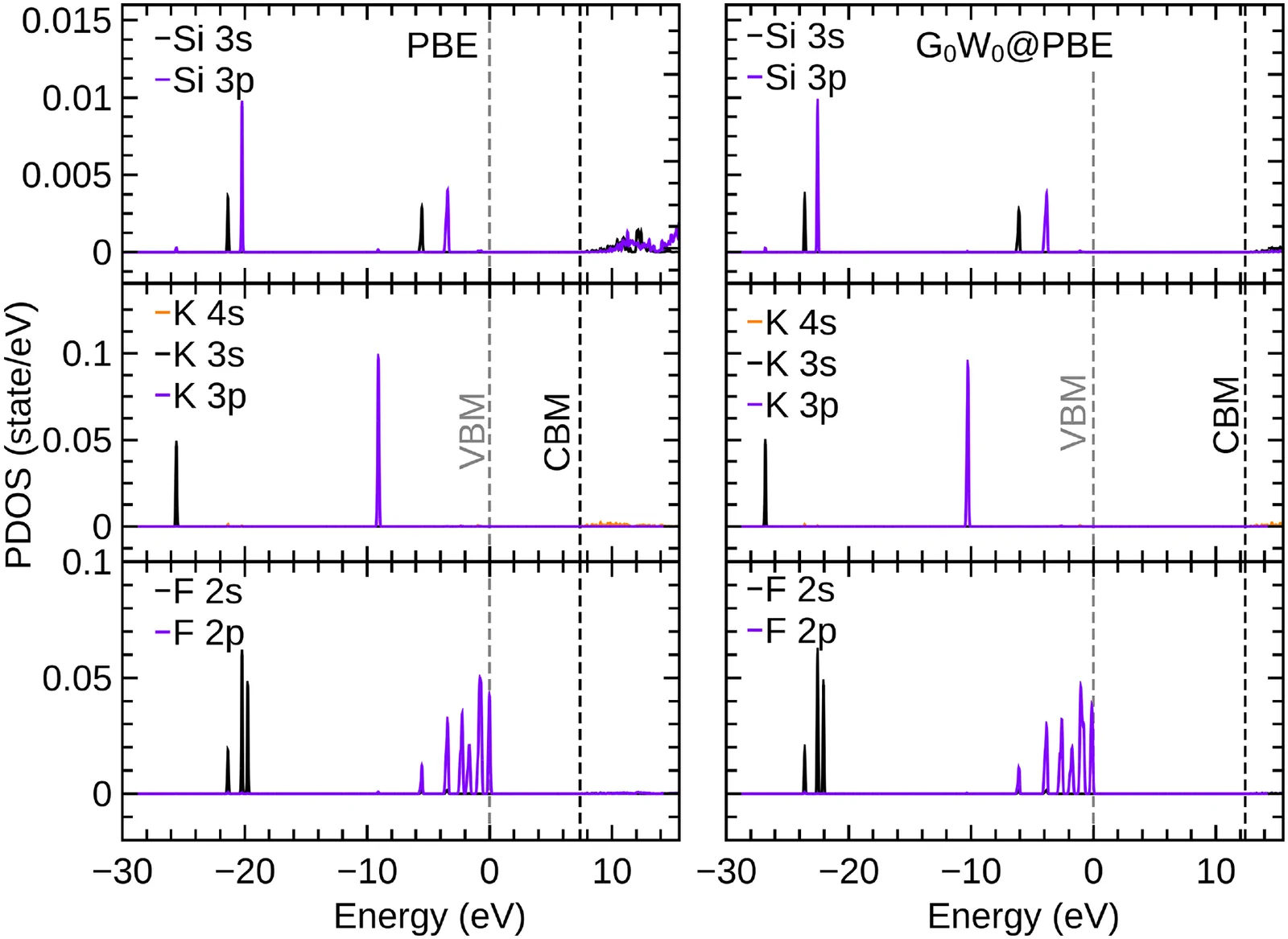
Orbital-projected density of states (pDOS) of K_2_SiF_6_ calculated at the PBE (left panels) and G_0_W_0_ (right panels) levels. The valence band maximum (VBM) is aligned to 0 eV in both cases. Grey and black dashed lines indicate the VBM and conduction band minimum (CBM), respectively.

The densities of states (DOS) calculated at the PBE and G_0_W_0_@PBE levels, as well as the orbital-projected densities of states (pDOS) are shown in Fig. 3 and 4. Besides the increase in the band gap, we find that the DOS and pDOS curves predicted by PBE and G_0_W_0_ are qualitatively similar, with G_0_W_0_ shifting the deeper valence and semi-core states to more negative energies. In agreement with previous work,^22^ we find that the states near the top of the valence band are almost exclusively derived from F 2p atomic orbitals, whereas both the K 4s and Si 3p orbitals contribute near the bottom of the conduction band. The pDOS curves indicate that the K–F bonds are highly ionic: the K 3s and K 3p orbitals only contribute to narrow atomic-like bands at approximately −10 eV and −26 eV, with almost no hybridization with F and Si orbitals, whereas the K 4s orbitals only contribute to the conduction band.

The highly ionic character of the bonding is corroborated by the Bader charge analysis shown in Table 2. The Bader charge on K, +0.91*e*, is close to the formal oxidation state of +1, indicating that K_2_SiF_6_ can be thought of as an ionic solid consisting of K^+^ cations and molecular [SiF_6_]^2−^ anions.

**Table 2:** Comparison of the valence charge, *Z*_val_, and the electron population, *N*_Bader_, from a Bader charge analysis in K_2_SiF_6_

Atom	*Z*_val_	*N*_Bader_	*q*_eff_ = *Z*_val_ − *N*_Bader_
Si	4	0.82	+3.18
K	9	8.09	+0.91
F	7	7.83	−0.83

**Fig. 5:**
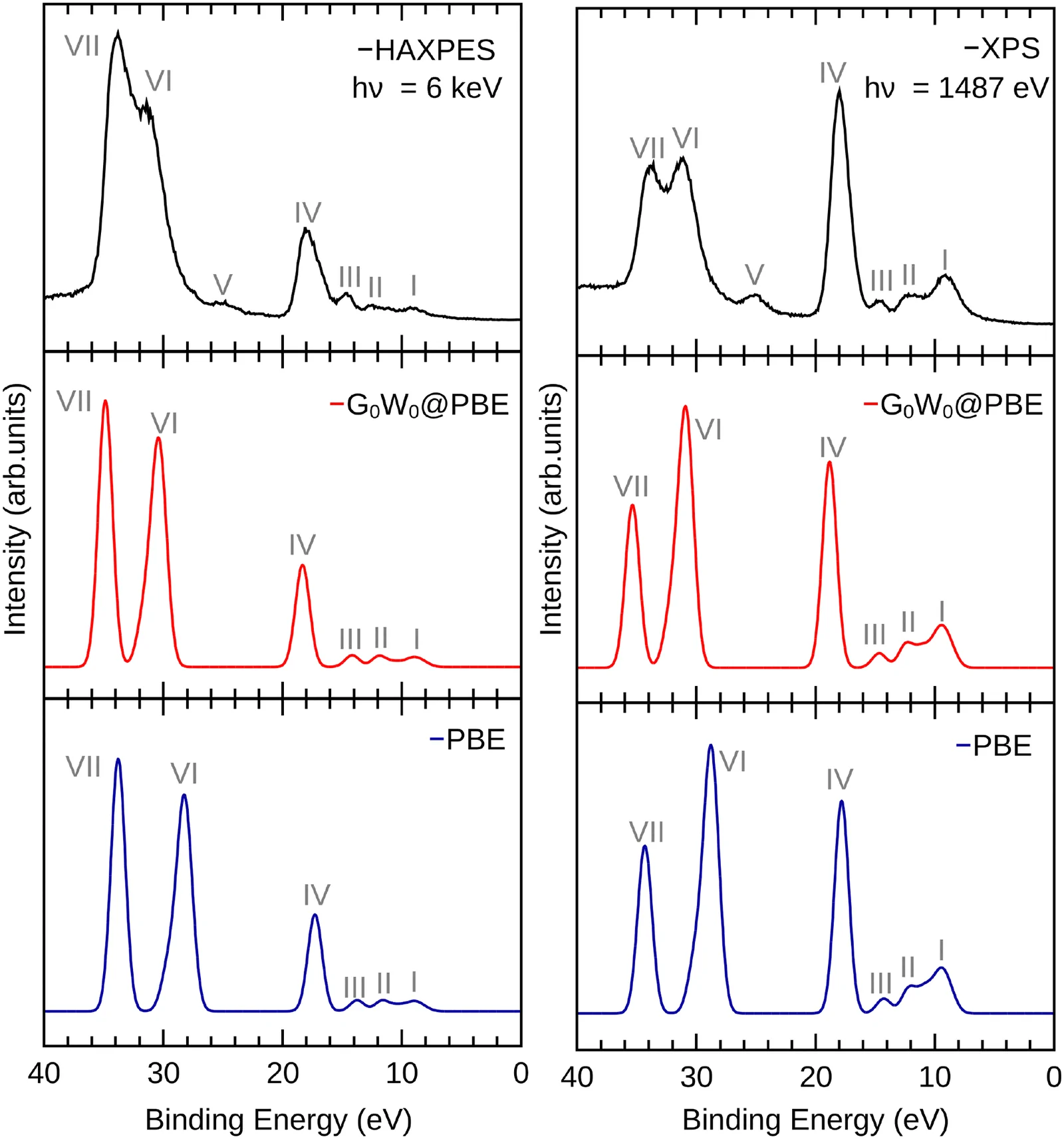
Comparison of experimental HAXPES (left panel) and XPS (right panel) valence band spectra of K_2_SiF_6_ with calculated spectra from PBE and G_0_W_0_@PBE. The calculated spectra have been constructed as cross-section weighted densities of states, with 1.4 eV of uniform Gaussian broadening. Experimental spectra were aligned to the respective C 1s spectra at a BE of 284.8 eV. Calculated spectra have been shifted such that peak I at ∼9 eV is aligned with the respective peak in the experimental spectrum.

The experimental valence-band photoelectron spectra of K_2_SiF_6_, recorded using HAXPES (*hν* = 6 keV) and Al Kα XPS (*hν* = 1487 eV), are compared with calculated spectra in Fig. 5. The theoretical spectra are constructed within the Gelius approximation^45^ as cross-section–weighted sums of the orbital-projected densities of states, using photoionization cross-sections from Scofield^46^ at 6 keV and from Yeh and Lindau^47^ at 1487 eV. For direct comparison, the calculated spectrum must be manually aligned with the experimental one, because in the real sample with defects, the position of the Fermi level within the band gap is not known *a priori*. In Fig. 5 the calculated spectra are shifted such that the first valence band peak at approximately 9 eV is aligned with the corresponding peak in the experimental spectrum. Overall, the calculated spectra from both the PBE and G_0_W_0_ calculations are in good agreement with experiment.

In the HAXPES spectrum, peak VII at ∼34 eV can be assigned to the K 3s level. Peak VI at ∼31 eV corresponds to F 2s levels hybridized with the Si 3s and 3p orbitals, and peak IV at ∼18 eV is assigned to K 3p. Peaks VI and VII exhibit higher intensity than the K 3p feature (peak IV) due to the larger photoionization cross-sections of the F 2s and K 3s orbitals at hard X-ray photon energies. At lower binding energies, several weaker features – peaks I–III – originating from the valence band dominated by F 2p states are observed. Amongst those, the strongest one is feature III at ∼14 eV – the spectral weight of this peak is higher in HAXPES, relative to XPS, due to the contribution of Si 3s orbitals – at 6 keV, the per-electron Si 3s photoionization cross-section is ∼24 times higher than the F 2p cross-section.^46^

Strong features corresponding to the K 3s, F 2s and K 3p levels (peaks VII, VI, and IV) are also visible in the XPS spectrum, but with relative intensities that differ from those observed in HAXPES due to the photon-energy dependence of photoionization cross-sections. In addition, in XPS, feature III at ∼14 eV is not as pronounced relative to the rest of the valence band, since the Si 3s contribution is smaller – at 1487 eV, the Si 3s photoionization cross-section is only ∼4.3 times larger compared to F 2p.

Both the HAXPES and XPS spectra also exhibit an additional weak peak near 25 eV, labelled peak V, that is absent in the calculated spectra. This feature is assigned to oxygen impurities. The assignment is supported by the XPS and HAXPES survey spectra shown in the SI. The survey spectra contain the expected peaks for K, Si, and F, and also indicate the presence of adventitious carbon, small amounts of Na and Mg impurities, and some oxygen. Based on analysis of the relative intensities of the F 1s and O 1s peaks and the relevant photoionization cross-sections we find that the F to O ratio is approximately 5.2 : 1 in the region probed by XPS, and 16 : 1 in the region probed by HAXPES. Since XPS is more surface sensitive than HAXPES, we conclude that oxygen is predominantly present at the surface of the material.

**Fig. 6:**
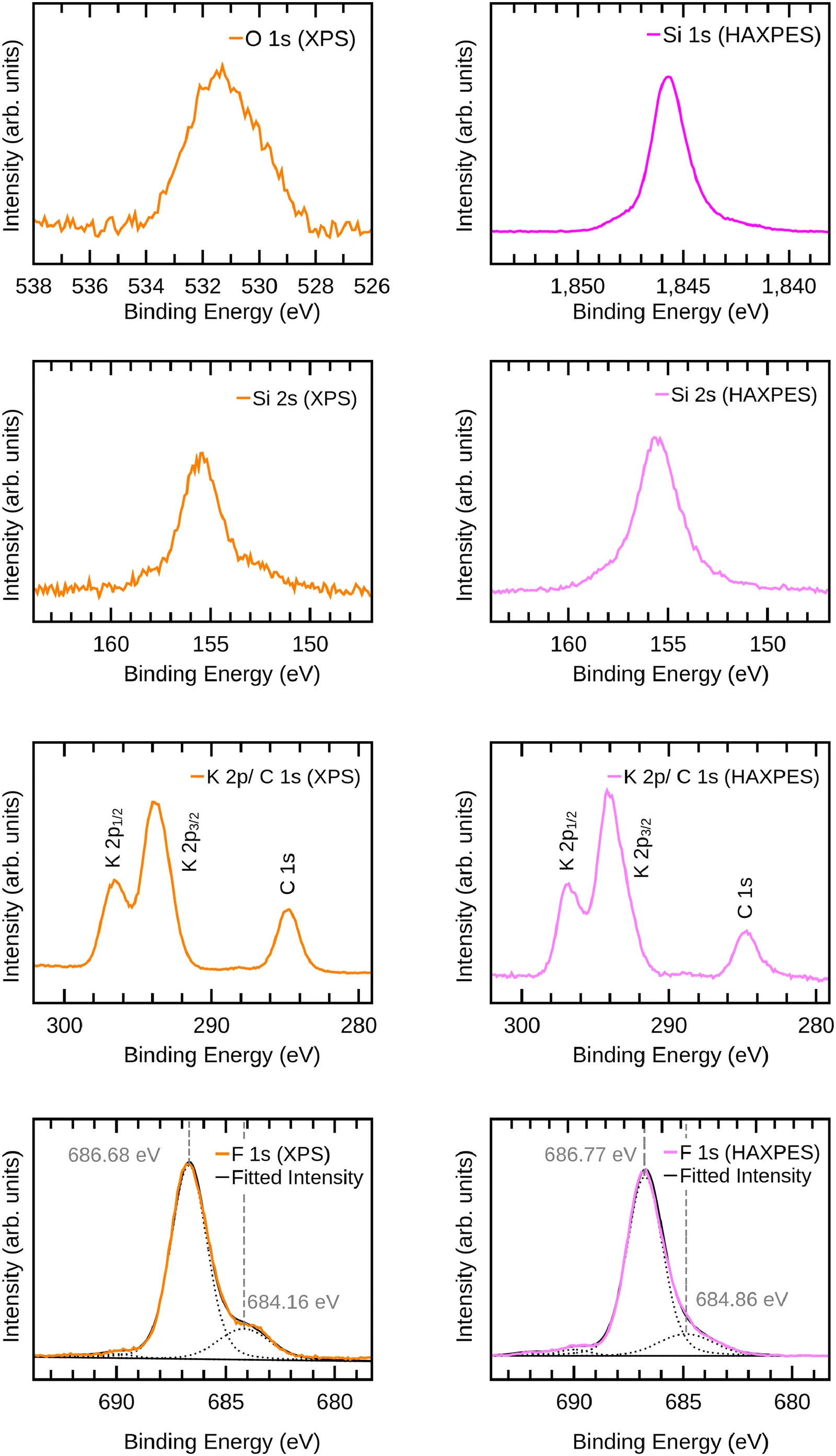
Core-level photoelectron spectra of K_2_SiF_6_ obtained using XPS (left panels) and HAXPES (right panels), showing the F 1s, K 2p/C 1s, Si 2s, Si 1s, and O 1s regions. All spectra were aligned to the respective C 1s spectra at a BE of 284.8 eV.

Core level photoelectron spectra of K_2_SiF_6_ are shown in Fig. 6. The F 1s core level spectra also indicate the presence of a different F chemical environment in the surface layer. In particular, the main peak at ∼686.7 eV in both the XPS and HAXPES spectra is assigned to the bulk material, whereas a secondary low-energy shoulder is considered a surface component as it is considerably more pronounced in the XPS spectrum than in the HAXPES spectrum. The binding energy of the surface component is 684.16 eV in the XPS spectrum. However, it was not possible to obtain a satisfactory fit of the HAXPES spectrum if the same binding energy of the surface component was enforced. One can speculate that this indicates inhomogeneities in the partly oxygenated surface layer. That said, we emphasize that the detailed analysis of core level lineshapes in this material is complicated by the strongly insulating character of the sample – the distortion of the lineshapes due to differential charging of the measurement region cannot be ruled out. The Si 1s, Si 2s, K 2p, and C 1s core level spectra are also included in Fig. 6 for completeness. The peak positions indicating the measured core electron binding energies are summarized in Table 3, and the details of the F 1s fits are provided in the SI.

**Table 3:** Experimental core level binding energies (BEs) in K_2_SiF_6_. All spectra were aligned to the respective C 1s spectra at a BE of 284.8 eV. The estimated error in BE position is ± 0.2 eV

Core level	Binding XPS	Energy (eV) HAXPES
F 1s	686.7	686.8
K 2p_3/2_	293.7	293.9
K 2p_1/2_	296.6	296.8
Si 2s	155.5	155.5
Si 1s	—	1845.7

## 4 Discussion

**Fig. 7:**
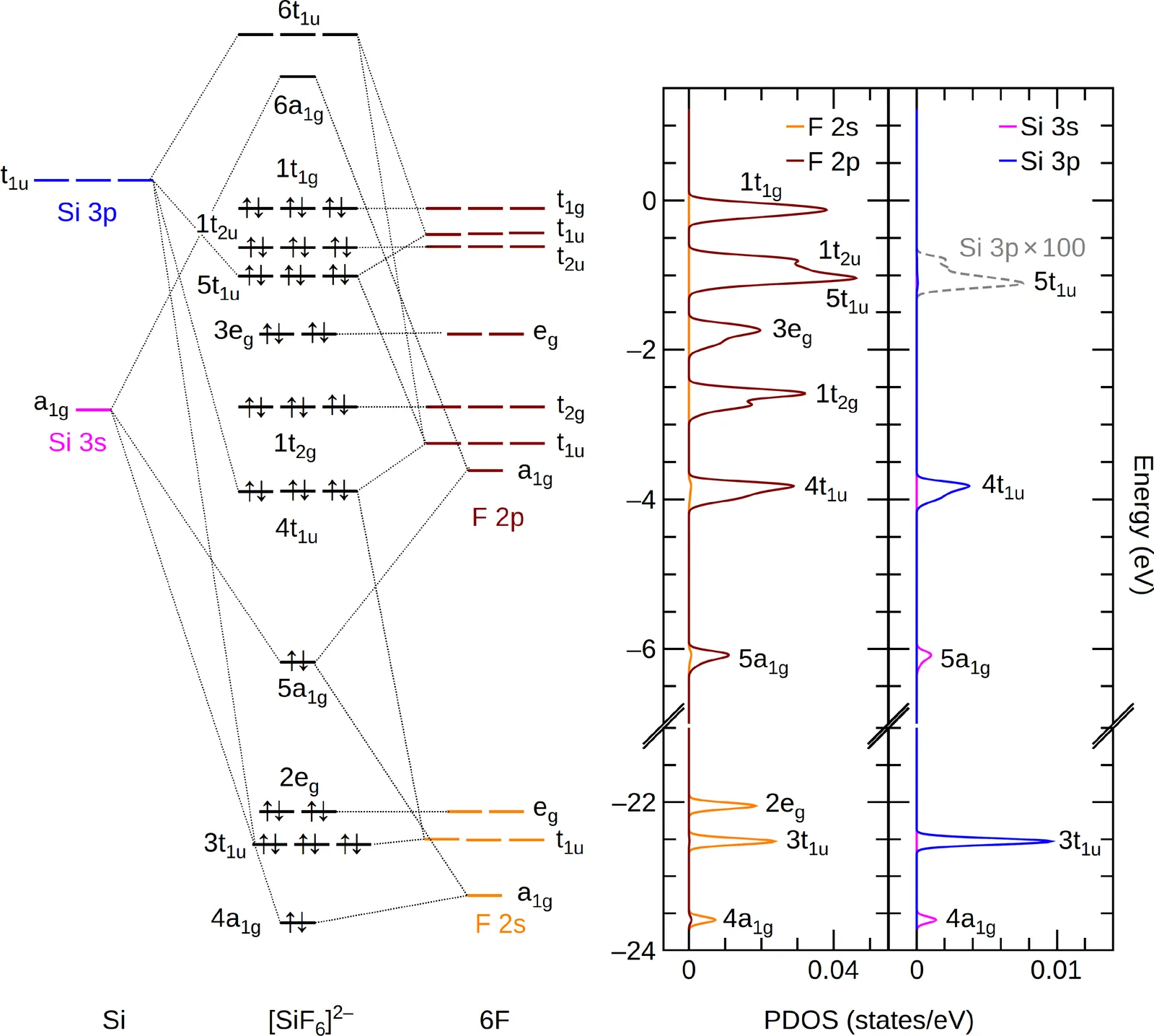
Molecular-orbital diagram of the isolated [SiF_6_]^2−^ unit and the corresponding G_0_W_0_@PBE projected density of states of K_2_SiF_6_.

The simplest way to describe the crystal structure of K_2_SiF_6_ is in terms of isolated [SiF_6_]^2−^ octahedra that are surrounded by K^+^ cations. The Bader charges given in Table 2 and the pDOS curves shown in Fig. 4 indicate that this partitioning is also appropriate for the electronic structure. Bonding within one [SiF_6_]^2−^ unit has a significant degree of covalent character, whereas bonding between the [SiF_6_]^2−^ octahedra and K^+^ cations is strongly ionic. With that in mind, the electronic structure of K_2_SiF_6_ can be further dissected by analyzing a single [SiF_6_]^2−^ unit using molecular orbital theory. [SiF_6_]^2−^ is isoelectronic with the SF_6_ molecule, and the corresponding molecular orbital (MO) diagram with symmetry labels corresponding to the irreducible representations of the octahedral (*O*_h_) point group can be found in many chemistry textbooks. This MO diagram is also shown in Fig. 7, together with the pDOS curves for Si and F from this work. Importantly, in the *O*_h_ point group, the Si 3s orbital can only contribute to states of a_1g_ symmetry, and the three Si 3p orbitals transform together as t_1u_, and only contribute to t_1u_ molecular orbitals.

Fig. 7 shows that there is a direct one-to-one correspondence between the features of the pDOS curves and the molecular orbitals predicted by group theory. In particular, each well resolved peak in the pDOS corresponds to a different MO, with the one exception that the 5t_1u_ and 1t_2u_ levels are so close in energy that they appear as one peak in the density of states. As dictated by symmetry, the Si 3s pDOS is nonzero only for the a_1g_ MOs, and the 3p pDOS is only nonzero for the t_1u_ MOs. In a periodic solid like K_2_SiF_6_, a molecular orbital picture is naturally an approximation – in the present case, it corresponds to the complete neglect of hybridization between K and the other elements. Nonetheless, the high level of agreement between the MO diagram and the pDOS curves confirms that in K_2_SiF_6_, nearest-neighbour interactions between Si and F atoms dominate, and band formation is a much weaker secondary effect. It should be noted that in contrast to the valence band, in the conduction band, wide bands are formed due to the participation of the spatially diffuse K 4s orbitals.

The results of our calculations also allow us to consider, whether K_2_SiF_6_ is a good candidate for a cross-luminescence based X-ray scintillator. Cross-luminescence in K_2_SiF_6_ is probable only if Auger–Meitner decay of semi-core holes is suppressed. In a simple picture, Auger–Meitner decay depends on the maximum energy that can be gained, when a valence electron drops down to fill a K 3p hole, and the minimum energy, that is required to promote a valence electron to an unbound empty state. The first quantity can be approximated as the difference between the K 3p level and the VBM, whilst the second quantity corresponds to the fundamental or quasiparticle gap in K_2_SiF_6_, *i.e.* the eigenvalue difference between the VBM and the CBM. If the K 3p-to-VBM difference is greater than the quasiparticle gap, Auger–Meitner decay is allowed. If it is smaller, Auger–Meitner decay is forbidden.

The K 3p-to-VBM energy separation is between 9 and 10 eV according to the valence band photoelectron spectra. A value of ∼9 eV is also produced by PBE, and G_0_W_0_ predicts this energy difference to be ∼10 eV. When comparing this value to the band gap, we see that according to PBE, the band gap is significantly smaller, and Auger-Meitner decay of K 3p holes should be allowed. For the band gaps predicted by regular hybrid functionals (B3LYP, PBE0, B1WC – 10.03, 10.84, 9.83 eV),^25^ the situation is borderline, whereas for G_0_W_0_ the calculated band gap of 12.4 eV clearly exceeds the K 3p – VBM separation, meaning that Auger–Meitner decay of K 3p holes is not possible.

Since the G_0_W_0_ fundamental gap is likely the most accurate, we conclude that a different mechanism must be responsible for the very low luminescence yields reported experimentally for K_2_SiF_6_.^8^ For example, if the optical gap in K_2_SiF_6_ is significantly smaller than the fundamental gap, which is typical for wide band gap insulators, K 3p holes could still be quenched by energy transfer to an exciton. Defect states inside the band gap of K_2_SiF_6_ may also play a role, especially as the presence of oxygen in the surface region of the material has been confirmed by XPS and HAXPES. Finally, multi-phonon scattering is another possible nonradiative decay channel for K 3p holes.

We also note that our conclusions regarding Auger–Meitner decay are in principle sensitive to the specific “flavour” of the GW approximation that is used. Issues such as the dependence of calculated band gaps on the method used for treating the frequency dependence of the dielectric matrix, or the degree of self-consistency in the GW calculation, have been discussed extensively in the scientific literature.^48–56^ The framework used in this study that combines one-shot GW, *i.e.* G_0_W_0_, with the Hybertsen–Louie plasmon-pole approximation, was chosen as it has been previously found to yield accurate band gaps in solids at a moderate computational cost.^48,49,56,57^

## 5 Summary

In this work, K_2_SiF_6_ has been studied using photoelectron spectroscopy, and DFT and GW theory. The experimental and theoretical results present a consistent picture of the valence band electronic structure of K_2_SiF_6_ that is dominated by nearest-neighbour interactions within a single [SiF_6_]^2−^ octahedron. K atoms contribute to atomic-like 3s and 3p occupied states, and bonding between K^+^ and [SiF_6_]^2−^ units has a strong ionic character. In contrast, in the conduction band, the diffuse K 4s orbitals are strongly hybridized with orbitals centered on Si to yield highly dispersive bands. The fundamental band gap of K_2_SiF_6_ predicted by G_0_W_0_ calculations is 12.4 eV. Comparison of the quasiparticle gap predicted by G_0_W_0_ with the K 3p-to-VBM separation suggests that Auger–Meitner decay of K 3p holes should be suppressed in K_2_SiF_6_, which is a necessary condition for cross-luminescence based scintillation. The fact that nonetheless, the experimentally observed light yield for cross-luminescence is very low, therefore points towards the importance of factors that were not considered in the present study. Excitonic effects, final-state relaxation, possible decay channels involving defect states or surface states, and multi-phonon scattering are highlighted as some of the aspects that deserve further attention.

## Author contributions

UK performed the DFT and GW calculations, prepared the figures and tables, and contributed to writing the manuscript. SL, PB, AAR, PKT, TLL, and AR performed the experimental photoelectron spectroscopy measurements. SL and AR also participated in the analysis of the XPS and HAXPES data, and contributed to writing the manuscript. TK performed preliminary XPS measurements to assess the suitability of the K_2_SiF_6_ sample for a HAXPES study, and also participated in the analysis of the results. MK and VN proposed the study, and contributed to the analysis of the results in light of the performance of K_2_SiF_6_ as a scintillator material. JL supervised the computational work and contributed to writing the manuscript. JMK supervised the computational work and the molecular orbital based analysis, coordinated the theory-experiment collaboration, and contributed to writing the manuscript. All authors read and approved the final manuscript.

## Conflicts of interest

All authors declare no financial or non-financial competing interests.

## Supplementary Material

TC-014-D6TC01537A-s001

## Data Availability

The datasets generated and analyzed during the current study are available in the Zenodo repository.^58^ Supplementary information (SI): (i) analysis of the F to O ratio in the sample, (ii) justification for the assignment of feature V in the valence band spectra, (iii) numerical details of the F 1s core level peak fits, (iv) XPS and HAXPES survey spectra, (v) numerical convergence of the GW results. See DOI: https://doi.org/10.1039/d6tc01537a.
