# Supplementary material for: The electronic structure of K2SiF6 from GW calculations and photoelectron spectroscopy
Source: J Mater Chem C Mater. 2026 Jul 17;14(34):15522–30. doi: 10.1039/d6tc01537a (PMC13563437; doi:10.1039/d6tc01537a)
Supplement: Supplementary file 1 [file TC-014-D6TC01537A-s001.pdf]

Supplementary Information (SI) for Journal of Materials Chemistry C.
This journal is © The Royal Society of Chemistry 2026

Supplementary Information for

"The Electronic Structure of K_2SiF_6 from GW Calculations and Photoelectron Spectroscopy"

Uday Kushwah, (Ann) Shiyang Lu, Prajna Bhatt, Aysha A. Riaz, Pardeep K. Thakur, Tien-Lin Lee, Marco Kirm, Vitali Nagirnyi, Tanel Käämbre, Johannes Lischner, Anna Regoutz, and Juhan Matthias Kahk

March 2026

1. Analysis of the F to O ratio based on the survey spectra

In order to quanitfy the amount of oxygen in the near-surface region of the sample, a simple analysis based on integrated peak areas from the survey spectra recorded at 6 keV and 1487 eV was carried out.

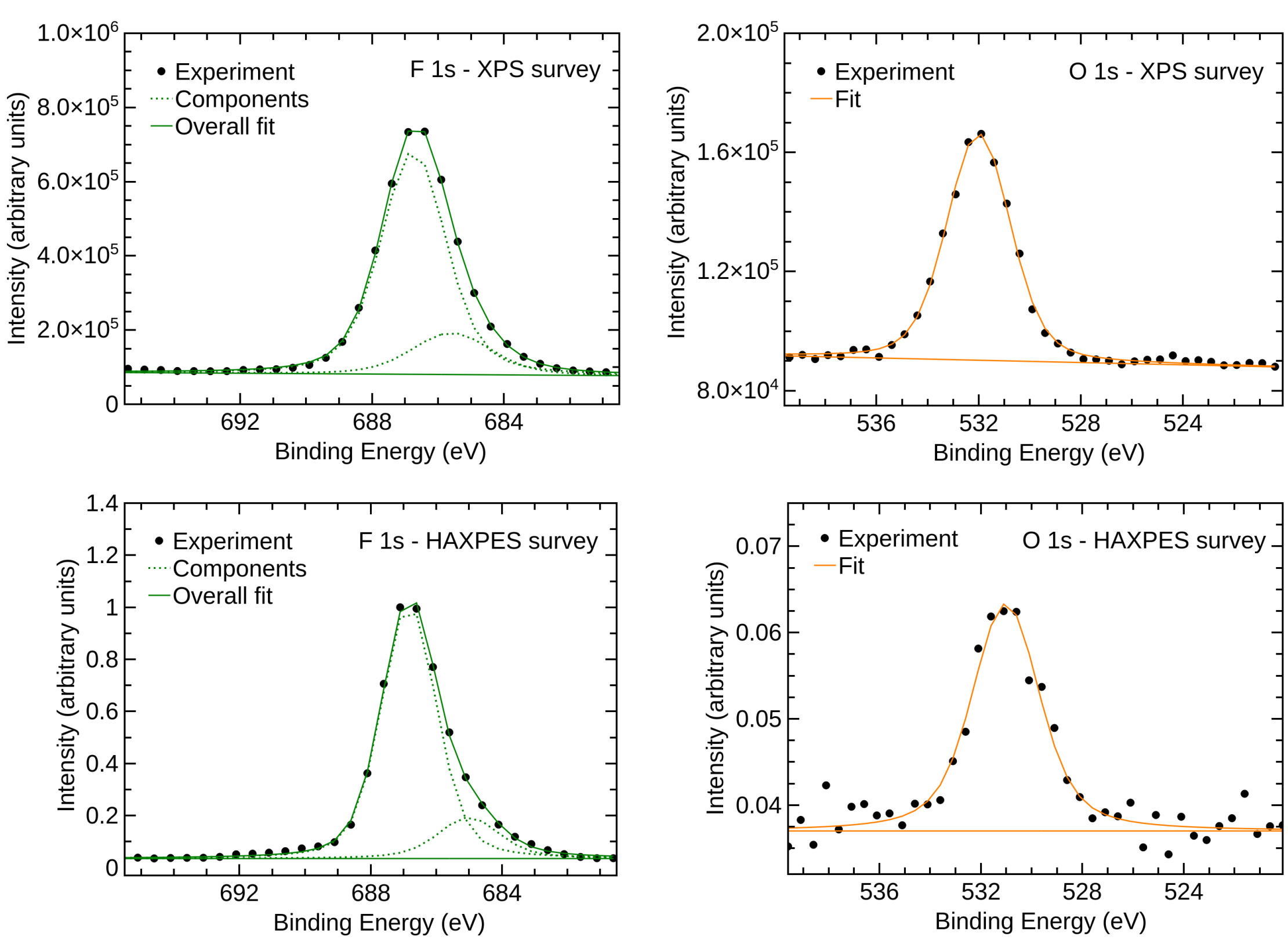

	HAXPES (hν = 6 keV)	XPS (hν = 1487 eV)
F 1s area (first component)	2.318	1717200
F 1s area (second component)	0.443	379500
F 1s total peak area	2.761	2096700
O 1s total peak area	0.106	266500
F 1s to O 1s area ratio	26.05	7.87
F 1s cross-section	1110 (ref 39)	0.06041 (ref 40)
O 1s cross-section	682.05 (ref 39)	0.04005 (ref 40)
F 1s to O 1s cross-section ratio	1.63	1.51
**F to O element ratio**	**16.0**	**5.22**

* the absolute peak areas are in arbitrary units – only the area ratios are meaningful

In summary, we find that the F to O ratio is ~16 in the region probed by HAXPES, and ~5.2 in the region probed by XPS, indicating that oxygen is mostly present in the surface layer. We note that two components were used for F 1s purely for obtaining a good fit for quantification purposes. The actual F 1s lineshapes, as visible in the high-resolution F 1s spectra, are discussed separately in the main article and in section 3 of this document.

2. Assignment of feature V in the valence band

Peak V in the extended valence band photoelectron spectrum at ~ 25 eV does not match any of the features in the calculated spectra. The binding energy suggests that it likely corresponds to the O 2s level of the oxygen impurity atoms. Below, the intensity of this feature is compared to the intensity of the F 2s peak, to ensure that this assessment is plausible, in light of the F to O ratios in XPS and HAXPES determined above. In order to obtain the peak areas of the O 2s and F 2s peaks, the extended valence band photoelectron spectra (XPS and HAXPES) have been fitted using Voigt lineshapes. This is naturally a crude approximation, as the true shape of the valence band density of states is neglected, but for the O 2s and F 2s levels it is deemed sufficient for an approximate quantification.

In K_2SiF_6, F 2s orbitals are hybridized with Si 3s and 3p orbitals, so some of the intensity of the "F 2s" photoemission peak (peak VI in Figure 6 of the main paper) comes from the Si 3s/3p contributions. However, since (i) the Si 3s contribution to the DOS is very small in that region, and (ii) the Si 3p photoionization cross-section is very low compared to F 2s, over 98% of the total intensity of peak VI is estimated to originate from F 2s orbitals, and in the analysis below, the contribution of Si 3s and 3p levels is ignored.

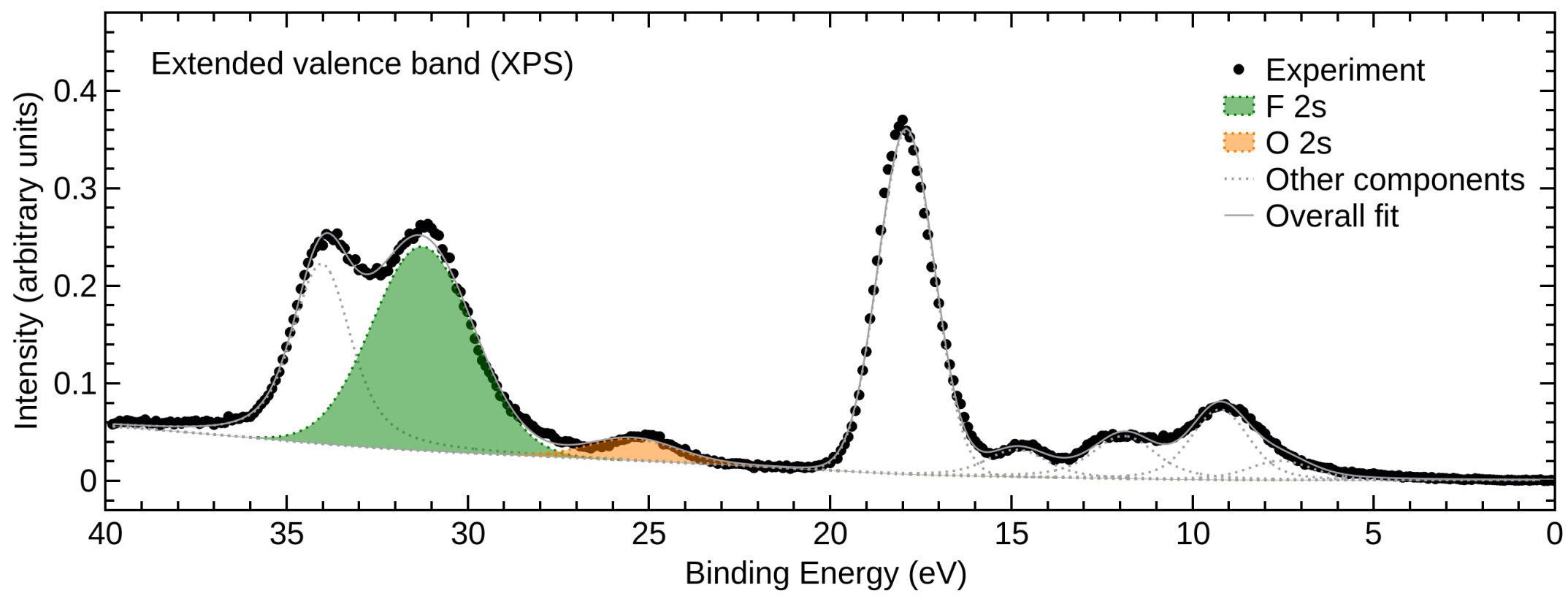

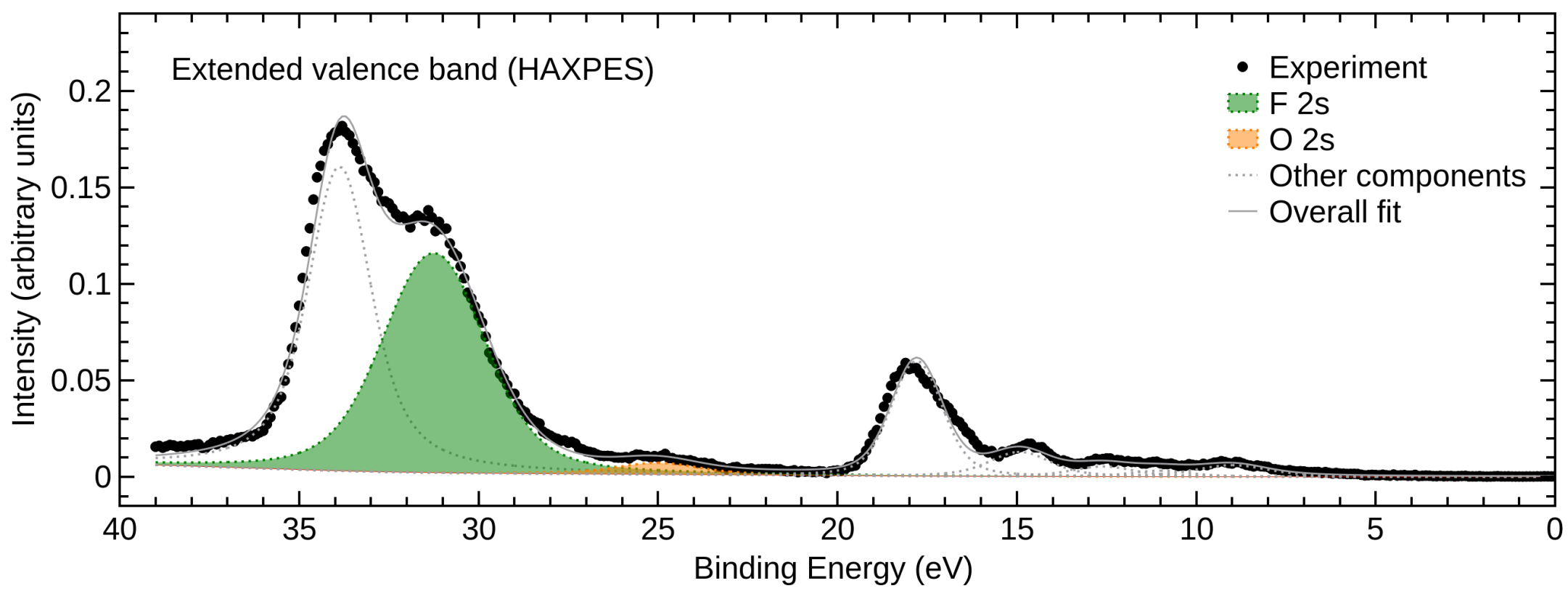

	HAXPES (hν = 6 keV)	XPS (hν = 1487 eV)
F 2s peak area	0.465	0.76
O 2s area	0.022	0.065
F 2s to O 2s area ratio	21.136	11.69
F 2s cross-section	59.71	0.002846
O 2s cross-section	36.59	0.001902
F 2s to O 2s cross-section ratio	1.632	1.4963
**F to O element ratio**	**12.95**	**7.81**

* the absolute peak areas are in arbitrary units – only the area ratios are meaningful

The F to O element ratios obtained from the relative intensities of peaks V and VI, assuming that peak V is assigned to O 2s, are 12.95 for HAXPES, and 7.81 for XPS. Considering the large uncertainties involved in fitting the extended valence band and the photoionization cross-sections, these values are reasonably close to the ratios of 16.0 and 5.22 obtained from the F 1s and O 1s peaks. It is noted that in XPS (hν = 1487 eV) the kinetic energies of valence electrons are appreciably bigger than the kinetic energies of core electrons, and hence the valence spectrum is less surface-sensitive than the bulk spectrum, which may partly explain why the F to O ratio obtained from the 2s orbitals is greater than the ratio obtained from the 1s orbitals. Overall, this analysis confirms the assignment of peak V to O 2s orbitals of impurity O atoms present mostly at the surface of the material.

3. F 1s peak fits

The numerical details of the F 1s peak fits shown in Figure 7 of the main paper are provided below. FWHM = full width at half maximum.

HAXPES peak fit				
	Peak 1	Peak 2	Peak 3	Peak 4
Lineshape	Voigt	Voigt	Voigt	Voigt
Centre	684.858	686.769	690.000	692.000
Amplitude	0.154	0.845	0.027	0.013
Lorentzian FWHM	0.183	0.197	0.170	0.170
Gaussian FWHM	1.179	0.727	0.650	0.650

XPS peak fit				
	Peak 1	Peak 2	Peak 3	Peak 4
Lineshape	Voigt	Voigt	Voigt	Voigt
Centre	684.158	686.681	690.000	692.000
Amplitude	7467.118	33296.143	703.040	280.000
Lorentzian FWHM	0.190	0.180	0.200	0.200
Gaussian FWHM	1.088	0.744	0.700	0.700

4. Survey spectra

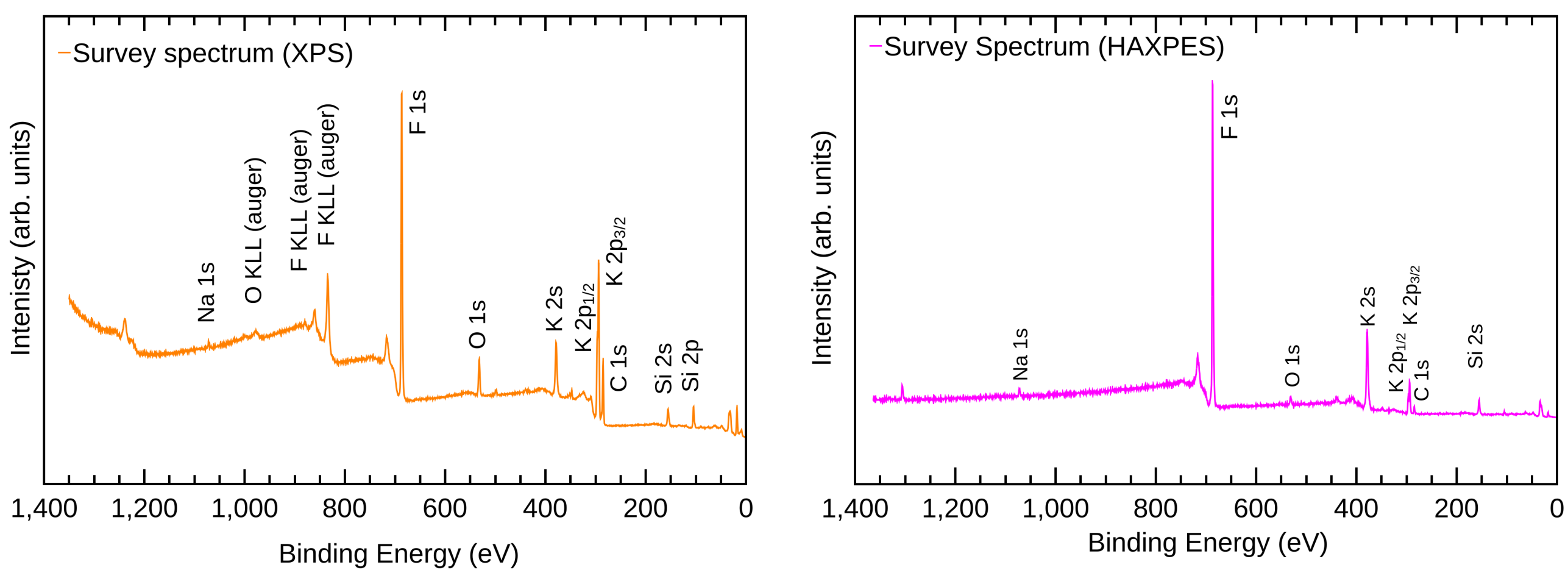

Survey photoelectron spectra of K_2SiF_6 measured at $h\nu = 1487$ eV (left) and $h\nu = 6$ keV (right).

5. Convergence and extrapolation of the GW eigenvalues

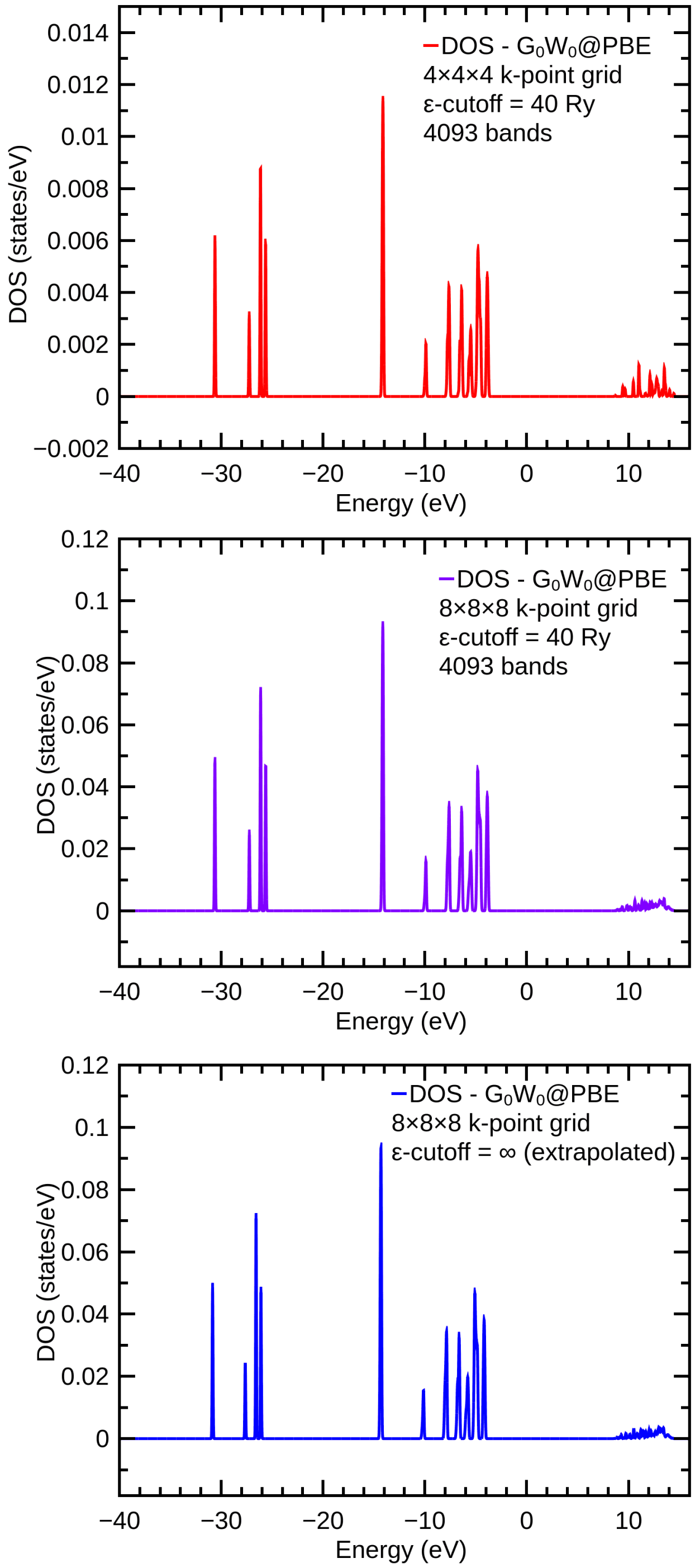

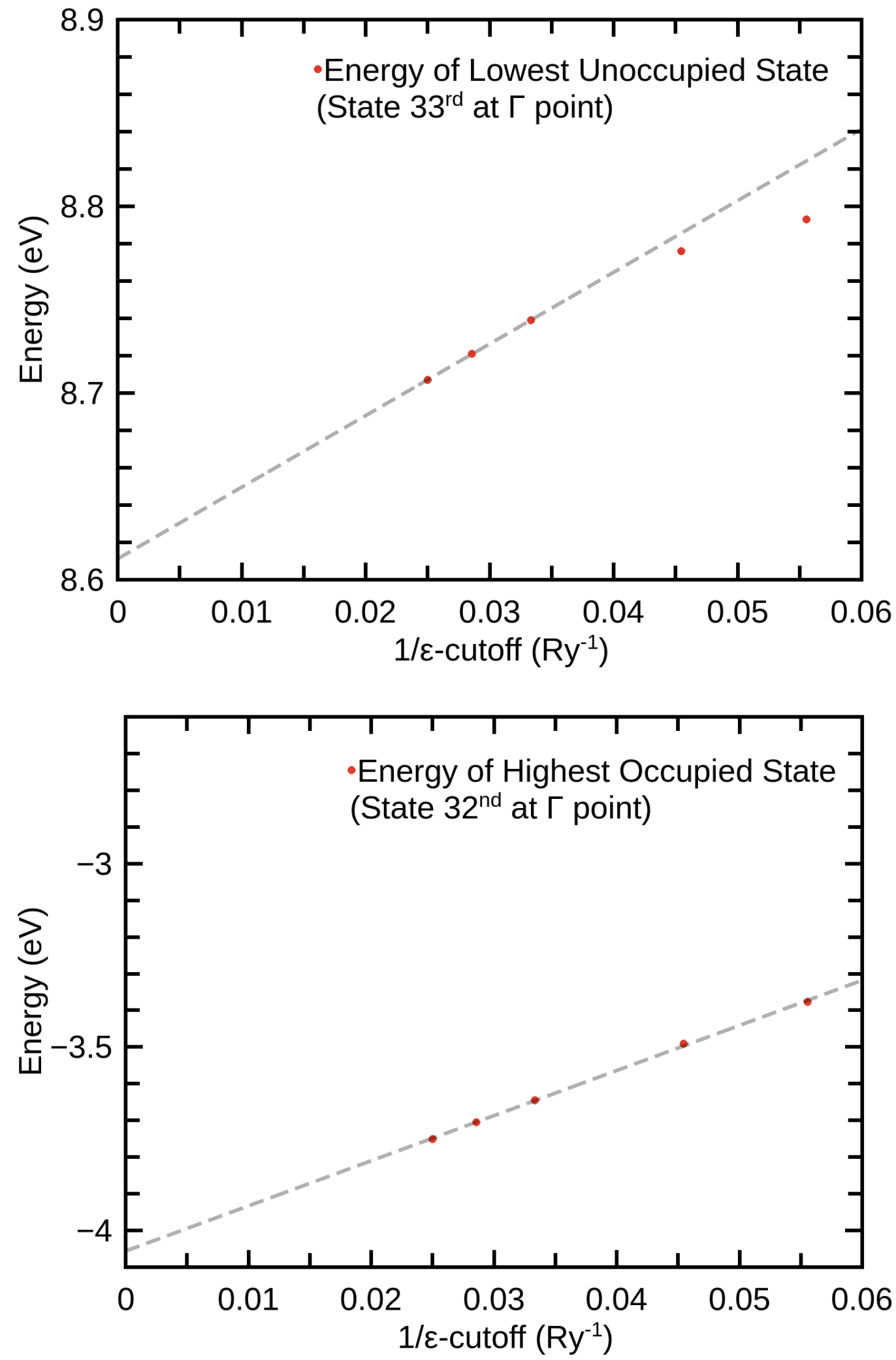

(Right panels) Extrapolation of the G_0W_0 eigenvalues to the infinite dielectric matrix cutoff (and infinite empty states) limit. The data are show for the CBM (top panel) and the VBM (bottom panel). Only the first three datapoints, corresponding to the three largest cutoff values, are used in the extrapolation.

(Left panels) The total density of states (DOS) in K_2SiF_6, calculated from the G_0W_0 eigenvalues.
Top: 4×4×4 k-point grid, dielectric matrix cutoff = 40 Ry, 4093 bands in the “sigma” calculation
Middle: 8×8×8 k-point grid, dielectric matrix cutoff = 40 Ry, 4093 bands in the “sigma” calculation
Bottom: 8×8×8 k-point grid, DOS constructed from the eigenvalues extrapolated to the limit of an infinite dielectric matrix cutoff and an infinite number of empty states
